# The Giant Panda Transferrin Receptor Facilitates Feline Parvovirus Infection to Drive Cross-Species Transmission

**DOI:** 10.3390/vetsci12070602

**Published:** 2025-06-20

**Authors:** Qigui Yan, Huanyuan Hu, Shan Zhao, Qin Zhao, Rui Wu, Xiaobo Huang, Yiping Wang, Yiping Wen, Yi Zheng, Fei Zhao, Sanjie Cao, Senyan Du, Yifei Lang

**Affiliations:** 1 College of Veterinary Medicine, Sichuan Agricultural University, Chengdu 611130, China; yanqigui@126.com (Q.Y.);; 2 Chengdu National Agricultural Science and Technology Center, Chengdu 610213, China

**Keywords:** feline parvovirus, transferrin receptor 1, virus replication, giant panda

## Abstract

Feline parvovirus (FPV) causes feline panleukopenia, a highly contagious disease in cats, marked by severe leukopenia, biphasic fever, diarrhea, vomiting, and hemorrhagic enteritis. Recently, FPV infection in giant pandas has increased, causing diarrhea and ultimately fatal outcomes, thereby threatening their survival and reproduction. Here, we investigated the transmission of FPV in giant pandas and its interaction with cellular receptors using an FPV strain (pFPV-sc) isolated from giant panda feces. Recombinant feline transferrin receptor 1 (fTfR1) and the giant panda ortholog (gpTfR1) were expressed in non-susceptible HEK293T and HeLa cells, while viral infection levels were measured to determine the effect of gpTfR1 on pFPV-sc replication. The findings indicated that gpTfR1 overexpression in non-susceptible cells significantly enhanced pFPV-sc replication, particularly influencing the viral attachment and internalization stages. Our data further revealed early-stage colocalization between gpTfR1 expression and virus infection, suggesting that gpTfR1 facilitates early viral infection and replication. Taken together, our study provides the first evidence on the mechanism of FPV cross-species infection in giant pandas and elucidates the interaction between gpTfR1 and FPV, which establishes a theoretical basis for the development of preventive and therapeutic strategies, thereby safeguarding the health and survival of giant panda populations from FPV.

## Simple Summary

Feline parvovirus (FPV) is a dangerous virus that causes severe illness in cats, including vomiting, diarrhea, and life-threatening infections. Recently, infection with this virus has also been reported in giant pandas, leading to similar symptoms and even death, putting these endangered animal species at risk. To understand how FPV spreads to pandas, we studied a panda-derived strain of the virus and examined how it interacts with panda host factors. The FPV virus uses a specific protein—namely the transferrin receptor 1—to enter and multiply inside cells. By testing this interaction in human cells that normally resist infection, we confirmed that the panda version of this receptor allows the virus to attach, enter, and replicate efficiently. These findings help explain why giant pandas are vulnerable to FPV and provide crucial insights for developing vaccines or treatments to protect them. Our research not only advances the understanding of cross-species virus transmission but also supports conservation efforts to safeguard giant pandas from deadly outbreaks.

## 1. Introduction

Feline parvovirus (FPV) causes a highly contagious acute infectious disease in cats named feline panleukopenia, with clinical symptoms characterized by severe leukopenia, biphasic fever, diarrhea, vomiting, and hemorrhagic enteritis [1]. FPV is characterized by its rapid evolution and spread. In addition to cats, cases of FPV cross-species transmission and infection in other animal species were frequent, such as deer, monkey, mink, tiger, leopard, lion, and panda, with isolates that retain over 95% of sequence homology across the viral genome, suggesting broad host adaptability despite conserved viral genetic architecture. Such infection often results in severe gastroenteritis, acute leukopenia, dehydration, lethargy, and high mortality, particularly in juveniles, due to the virus’s tropism for rapidly dividing cells located in the intestinal epithelium and hematopoietic tissues [2,3,4,5,6,7,8,9]. In recent years, FPV infection in giant pandas has become increasingly common, leading to diarrhea and, in severe cases, mortality. This poses a potential threat to the survival and reproduction of giant pandas and a substantial challenge to the conservation of endangered wildlife.

FPV belongs to the *Parvoviridae* family and *Parvovirus* genus. It is a non-enveloped, single-stranded DNA virus whose capsid has icosahedral symmetry and a diameter of approximately 25 nm. The full-length viral genome is approximately 5.1 kb and contains two major open reading frames. Through alternative splicing, the nonstructural proteins (NS1 and NS2) and capsid proteins (VP1 and VP2) are encoded by the same mRNA [10]. VP2 is the main capsid protein of FPV and contains many overlapping antigenic and binding sites for the host transferrin type 1 receptor (TfR1) [11,12]. TfR1 plays a key role in the immune responses and determines the viral ligand for host specificity and tissue tropism [13]. Although the VP2 sequences of different parvoviruses are very similar, they exhibit high host specificity, where even substitutions in one or two amino acids can contribute to host tropism. Previously, we isolated an FPV strain, pFPV-sc, from a group of infected giant pandas with mortality cases. Sequence analysis indicated 99.8% similarity between pFPV-sc and the closest FPV sequence, but pFPV-sc contains unique characteristics in its VP2 protein [8].

Virus-receptor interaction is the initiating step in the process of virus infection and replication and plays a key regulatory role in the virus–host range, tissue tropism, and virus pathogenesis. Studies have shown that FPV infection in feline kidney cells is mediated by binding to the apical domain of feline transferrin receptor 1 (fTfR1) [14]. Notably, the TfR1 protein sequences of different hosts differ in their apical domains, which directly affect the host range of parvoviruses. For instance, during canine adaptation, contemporary canine parvovirus (CPV) variants evolved an enhanced binding affinity for canine TfR1, increasing infectivity in dogs while concurrently exhibiting reduced binding efficiency to feline TfR1 compared to ancestral CPV strains, reflecting host-specific adaptive selection pressures [15]. To date, only a few studies have shown that FPV can infect giant pandas; however, the mechanism by which FPV infects giant pandas and the transmission and evolution processes in new hosts remain largely undetermined. Therefore, comprehensive research into the mechanisms of infection is imperative. In this study, we analyzed the effect of giant panda transferrin receptor 1 (gpTfR1) on pFPV-sc infection. Our data indicated that specific binding occurs between FPV and the gpTfR1 located on the cell surface, thereby promoting the virus to invade host cells and infect giant pandas. This discovery lays the foundation for an in-depth understanding of the mechanisms underlying cross-host infection by feline parvoviruses.

## 2. Material and Methods

### 2.1. Cells, Virus, and Antibodies

The pFPV-sc strain of feline parvovirus from giant pandas was previously isolated, identified, and preserved in our laboratory [8]. The feline kidney (F81), human embryonic kidney (HEK293T), and human cervical cancer (HeLa) cell lines were originally purchased from ATCC (American Type Culture Collection, Manassas, VA, USA) and preserved in our laboratory.

The antibodies used in the study included Anti-V5 Tag mouse monoclonal antibody (ABT2170, Abbkine, Wuhan, China), Anti-Myc Tag mouse monoclonal antibody (ABT2060, Abbkine, Wuhan, China), DyLight 488, Goat anti-Mouse IgG (A23210, Abbkine, Wuhan, China), fluorescein isothiocyanate (FITC) Goat anti-Rabbit IgG H&L (ab6717, Abcam, Cambridge, UK), and Fluor647-conjugated goat anti-mouse IgG (A-21240, Life Technologies, Frederick, MD, USA).

### 2.2. Sequence Analysis and Plasmid Construction

Primers were designed for the CD region of F81 *TfR1* (GenBank accession number: NM001009312) (Table 1). Total RNA was extracted from the F81 cells. After reverse transcription, the obtained cDNA was used as a template for polymerase chain reaction (PCR) amplification and ligated into the pcDNA3.1-v5/myc vector. The PCR amplification conditions were as follows: 95 °C for 3 min; 35 cycles of 95 °C for 15 s, 58 °C for 30 s, 72 °C for 40 s; and 72 °C for 5 min; and storage at 4 °C. Concurrently, with reference to GenBank: XM_034660038.1, the CD region of *gpfR1* was synthesized by Sangon Biotech (Shanghai) Co., Ltd. and connected to the vector pcDNA3.1-v5/myc. The successfully constructed plasmids were named pcDNA3.1-v5/myc-fTfR and pcDNA3.1-v5/myc-gpTfR1. The homology between the *fTfR1* and *gpTfR1* sequences was analyzed using Clustal Omega software (version 1.2.2).

### 2.3. Indirect Immunofluorescence Detection of pFPV-sc Infection in Different Cells

When the F81, HEK293T, and HeLa cells in the 24-well plate grew to approximately 90% confluence, the cells were washed twice with phosphate-buffered saline (PBS), and the pFPV-sc (multiplicity of infection [MOI] = 0.1) virus solution was inoculated. After 48 h of infection, the cells were fixed with 4% paraformaldehyde, and an indirect immunofluorescence experiment was performed. First, the cells were washed twice with PBS, fixed with 4% paraformaldehyde for 30 min, and washed twice with PBS, while the cell membrane was permeabilized and then blocked with 2% bovine serum albumin for 2 h. Subsequently, the cells were incubated with a rabbit anti-FPV immune serum (1:200 dilution) for 1 h, as described previously [8]. After removing the primary antibody solution, the cells were washed twice with PBS and incubated with FITC goat anti-rabbit IgG antibody diluted 1:200 for 1 h. The incubation solution was discarded and the cells were washed twice with PBS. Next, 4′,6-diamidino-2-phenylindole (DAPI) diluted 1:100 was added and incubated at room temperature for 10 min. The cells were then washed twice with PBS. Finally, the cells were mounted with Prolong Diamond (P36971, Life Technologies, MD, USA) according to the manufacturer’s instructions. The cells were observed and photographed using a fluorescence microscope.

### 2.4. qPCR Verification of TfR1 Overexpression and FPV Copy Number

HEK293T and HeLa cells were seeded in 12-well plates and incubated until 70% confluence for transient transfection. Next, using the Lipofectamine^TM^ 3000 (Life Technologies, USA) transfection method, 1 μg of pcDNA3.1-v5/myc-fTfR1 and pcDNA3.1-v5/myc-gpTfR1 plasmids was transfected. Concurrently, pcDNA3.1-v5/myc was used as a negative control (pcDNA3.1) and the untransfected blank group was used as a blank control (mock). Total RNA was extracted using the TRIzol reagent and reverse-transcribed. The obtained cDNA was used to detect overexpression of the recombinant plasmids using quantitative PCR (qPCR). The reaction mixture consisted of 2 × TB Green Premix Ex TaqTM II (RR820Q, Takara Biomedical Technology, Dalian, China) (10 μL); 0.5 pmol of upstream and downstream primers; and 7 μL of ddH_2_O; and 1 μL of cDNA. The reaction conditions were as follows: 95 °C for 30 s, 40 cycles of 95 °C for 5 s, and 60 °C for 30 s. The qPCR results were expressed using the 2^−ΔΔCT^ method, with β-actin serving as the endogenous control for normalization. The threshold cycle (CT) values of TfR1 were normalized to β-actin (ΔCT = CT_TfR1_ − CT_β-actin_), followed by calibration to a reference sample (ΔΔCT = ΔCT_sample_ − ΔCT_calibrator_). Relative TfR1 expression was calculated as 2^−ΔΔCT^.

After confirming that the overexpression was effective, and after infection with pFPV-sc (MOI = 0.1) for 36 h, the DNA of the viral solution was extracted, and the FPV copy number was determined by absolute quantification using a standard curve generated from serial 10-fold dilutions (10^7^–10^1^ copies/μL) of a linearized plasmid containing the FPV VP2 gene (Table 1). Cq values obtained from samples were interpolated from the standard curve, with copy numbers calculated using the formula [6.022 × 10^23^ × (concentration in ng/μL) × 10^9^]/(plasmid length × 660).

### 2.5. Indirect Immunofluorescence Verification of TfR1 Overexpression and pFPV-sc Replication Levels

HEK293T and HeLa cells were plated in 24-well plates containing circular coverslips. Next, 0.5 μg of pcDNA3.1-v5/myc-fTfR1 and pcDNA3.1-v5/myc-gpTfR1 plasmids were transfected. Concurrently, pcDNA3.1-v5/myc was used as a negative control (pcDNA3.1), and the untransfected blank group was used as a blank control (mock). After 36 h of transfection, the overexpression of *fTfR1* and *gpTfR1* was detected using an indirect fluorescence immunoassay. V5 and Myc-tagged mouse monoclonal antibodies were used as the primary antibodies, and DyLight 488 goat anti-mouse IgG was used as the secondary antibody. Finally, fluorescence was observed using a fluorescence microscope, and the localization of TfR1 was analyzed.

Concurrently, cells were inoculated with pFPV-sc (MOI = 0.1) for 36 h and fixed with 4% paraformaldehyde. Rabbit anti-pFPV-sc immune serum was used as the primary antibody, and FITC goat anti-rabbit IgG was used as the secondary antibody. Finally, fluorescence was observed using a fluorescence microscope to determine the number of FPV-infected cells.

### 2.6. Effect of gpTfR1 Expression on pFPV-sc Adsorption and Internalization

HEK293T and HeLa cells were inoculated with pFPV-sc (MOI = 0.5), incubated at 4 °C for 1 h, and washed twice with cold PBS to remove unadsorbed viruses. Next, three freeze–thaw treatments were performed to release the virus particles adsorbed on the cell surface. The supernatant was collected, viral DNA was extracted, and the viral Cq value in the adsorption stage was obtained using qPCR to calculate the FPV copy number. Concurrently, in a 24-well plate with a circular coverslip, the above procedure was repeated, the cells were fixed with 4% paraformaldehyde, and an indirect fluorescence immunoassay was performed to detect the viral fluorescence signals during the adsorption stage.

HEK293T and HeLa cells were inoculated with pFPV-sc (MOI = 0.5), incubated at 4 °C for 1 h, and then transferred to 37 °C and incubated for 1 h. The virus solution was removed and the unadsorbed viruses were removed by washing with pre-cooled PBS. Proteinase K solution (400 μL, 1 mg/mL) was added to each well to remove viruses bound to the cell surface. Three freeze–thaw treatments were performed to extract viral DNA and obtain the viral Cq value in the internalization stage using qPCR to calculate the FPV copy number. Concurrently, in a 24-well plate with a circular coverslip, after allowing internalization at 37 °C, the cells were immediately fixed with 4% paraformaldehyde at room temperature for 30 min. An indirect fluorescence immunoassay was performed to detect viral fluorescence signals during internalization. This procedure required the permeabilization of the cell membrane.

### 2.7. Colocalization Analysis of gpTfR1 Expression and pFPV-sc Infection

HEK293T and HeLa cells transfected with the recombinant plasmids were infected with pFPV-sc (MOI = 0.1) at 37 °C for 1 h. The cells were then thoroughly washed with PBS and fixed with 4% paraformaldehyde. Rabbit anti-FPV immune serum and the V5-tagged mouse monoclonal antibody were used as primary antibodies, and FITC-conjugated goat anti-rabbit IgG (green light) and Fluor647-conjugated goat anti-mouse IgG (red light) were used as secondary antibodies. Finally, the fluorescence signals were observed using a fluorescence microscope.

### 2.8. Examination of Cell Viability Detection by CCK8 Assay

The cell suspension was inoculated into a 96-well plate and incubated for 16 h. Plasmid transfection was performed for 36 h. After transfection, the cell supernatants were discarded. Next, 10 μL of the CCK8 solution and 90 μL of 10% Dulbecco’s modified Eagle’s medium were added to each well. After culturing for 2 h, the absorbance was measured at 450 nm using a microplate reader. The formula used is as follows: Cell viability (%) = (As − Ab)/(Ac − Ab) × 100%, where As is the OD value of the transfection experimental well, Ab is the OD value of the pcDNA3.1 empty vector-transfected well, and Ac is the OD value of the normal cell control well.

### 2.9. Data Analysis

All experiments were repeated at least three times. In the indirect immunofluorescence experiments, ImageJ software (version 1.52e) was used to count the infected cells. All statistical analyses were performed using unpaired two-tailed tests in GraphPad Prism version 9. Data are presented as the standard error of the mean of three independent experiments or replicates. Significance levels are as follows: ns, not significant; * *p* < 0.05; ** *p* < 0.01; and *** *p* < 0.001. Homology and amino acid site homology analyses of *fTfR1* (GenBank: NM001009312) and *gpTfR1* (GenBank: XM044382865) were performed using Clustal Omega software (version 1.2.2).

## 3. Results

### 3.1. Validation of Cell Tropism of the Giant Panda Derived FPV (pFPV-sc)

**Figure 1:**
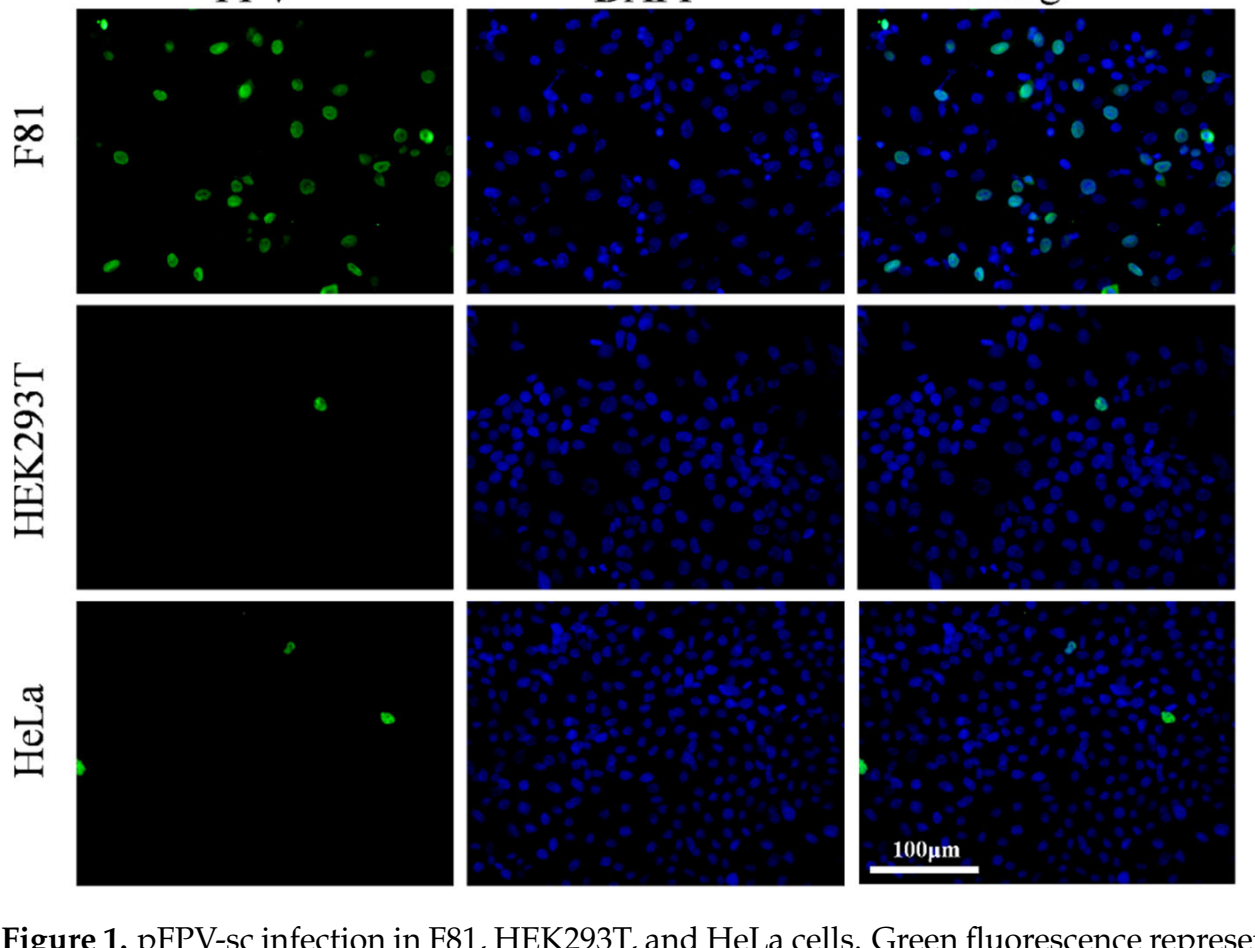
pFPV-sc infection in F81, HEK293T, and HeLa cells. Green fluorescence represents successful pFPV-sc infection.

Building upon previous observations, F81, HEK293T, and HeLa cells were selected for analysis of the cell tropism of pFPV-sc and the possibility of transducing exogenous TfR1 expression that might support pFPV-sc infection. Cells were inoculated with pFPV-sc at a multiplicity of infection [MOI] of 0.1, and indirect immunofluorescence was used to evaluate infection levels at 48 h post-infection. As shown in Figure 1, fluorescence signals were observed in F81 cells, whereas only a few fluorescence signals were observed in HEK293T and HeLa cells. This result indicates that pFPV-sc showed high susceptibility in F81 cells, whereas infectivity was relatively low in HEK293T and HeLa cells, which could be applied for examination for the effect of foreign TfR1 expression on virus infection.

### 3.2. Conformation of Expression of Recombinant fTfR1 and gpTfR1

**Figure 2:**
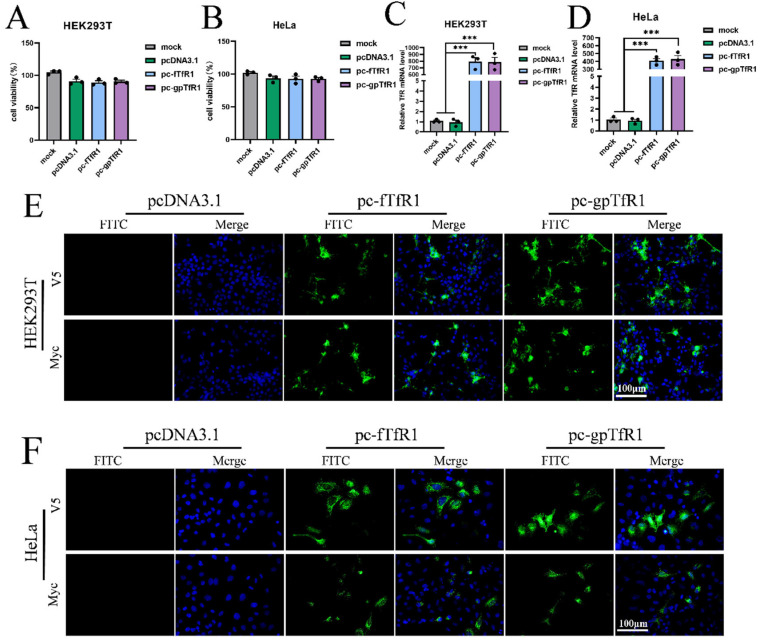
Expression identification of the recombinant fTfR1 and gpTfR1 plasmids. (**A**,**B**) CCK8 assay to detect cell viability after transfection with recombinant plasmids. (**C**,**D**) qPCR to detect the mRNA level of TfR1 in cells. (**E**,**F**) Indirect immunofluorescence assay against V5 or Myc tag to detect the expression of the fTfR1 and gpTfR1 overexpression vectors. (Blue: nucleus; Green: V5 or Myc tag; *** *p* < 0.001).

HEK293T and HeLa cells were transfected with pcDNA3.1-v5/myc-fTfR1 and pcDNA3.1-myc/v5-gpTfR1 recombinant plasmids, with pcDNA3.1-v5/myc empty plasmids as controls. The cell viability in each group was determined using the CCK8 method. The results showed that although cell viability in the cells transfected with *fTfR1* and *gpTfR1* slightly decreased, no significant difference compared to that of the mock and empty vector groups. Total RNA was extracted from each transfection group, and the *TfR1* mRNA content in each group of cells was measured. The results indicate that the *TfR1* mRNA content in the groups transfected with *fTfR1* and *gpTfR1* was significantly increased. In addition, using an indirect immunofluorescence assay, obvious fluorescence signals were observed in cells transfected with *fTfR1* and *gpTfR1*, whereas no fluorescence signal was observed in the control group (Figure 2). These results indicate that recombinant *fTfR1* and *gpTfR1* can be successfully induced in non-susceptible cells of pFPV-sc without altering cell viability.

### 3.3. Giant Panda TfR1 Promotes pFPV-sc Replication

**Figure 3:**
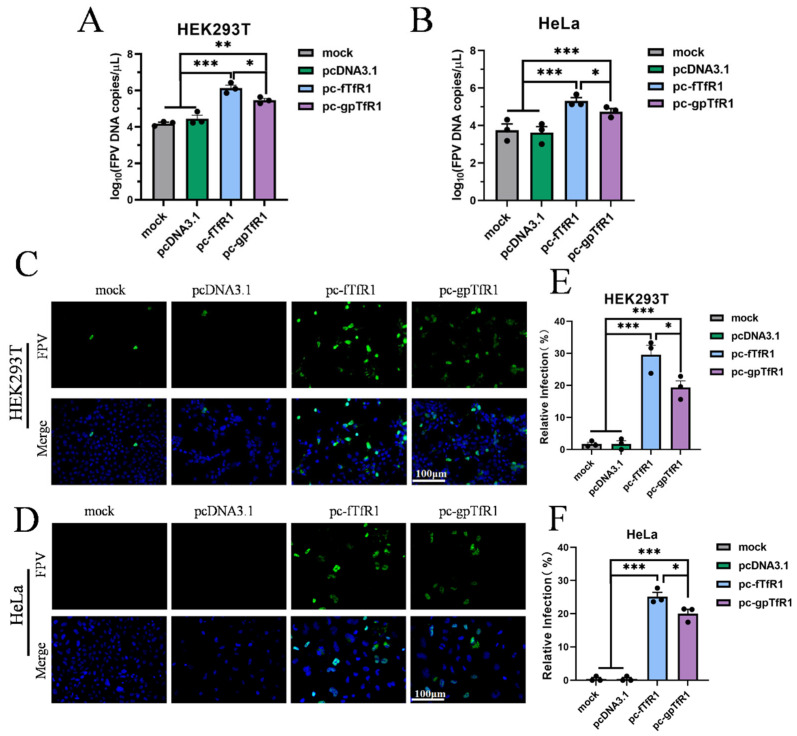
Overexpression of gpTfR1 promotes pFPV-sc replication. (**A**,**B**) qPCR to detect FPV copies. (**C**,**D**) Indirect immunofluorescence assay to detect the number of infected cells (Blue: nucleus; Green: FPV). (**E**,**F**) ImageJ counts of the percentage of infected cells. (* *p* < 0.05, ** *p* < 0.01, *** *p* < 0.001).

We next examined the probable effect of recombinant gpTfR1 on pFPV-sc infection in non-susceptible cells. Recombinant gpTfR1 or fTfR1 were expressed in HEK293T and HeLa cells prior to virus infection, and the results of qPCR analysis revealed that the viral copy number in the gpTfR1 group increased significantly in both cell lines in comparison with mock-transfected or empty vector-transfected cells (Figure 3A,B). Similar observations were obtained with indirect immunofluorescence analysis, where a strong fluorescence signal was observed in the gpTfR1 transfected group and the infection rate reached above 20% (Figure 3C–F). Differences were still observed in both the viral copy number and cell infection rate in the gpTfR1 group in comparison with the fTfR1 group, indicating a relatively lower viral replication (Figure 3). This may be due to the difference in amino acid sites between the two TfR1 receptors, resulting in different levels of receptor–virus interaction, where pFPV-sc still prefers fTfR1 over gpTfR1. Apparently, the expression of gpTfR1 is already favored by pFPV-sc, resulting in constructive infection in FPV-unsusceptible cells.

### 3.4. Overexpression of gpTfR1 Promotes pFPV-sc Adsorption and Internalization

**Figure 4:**
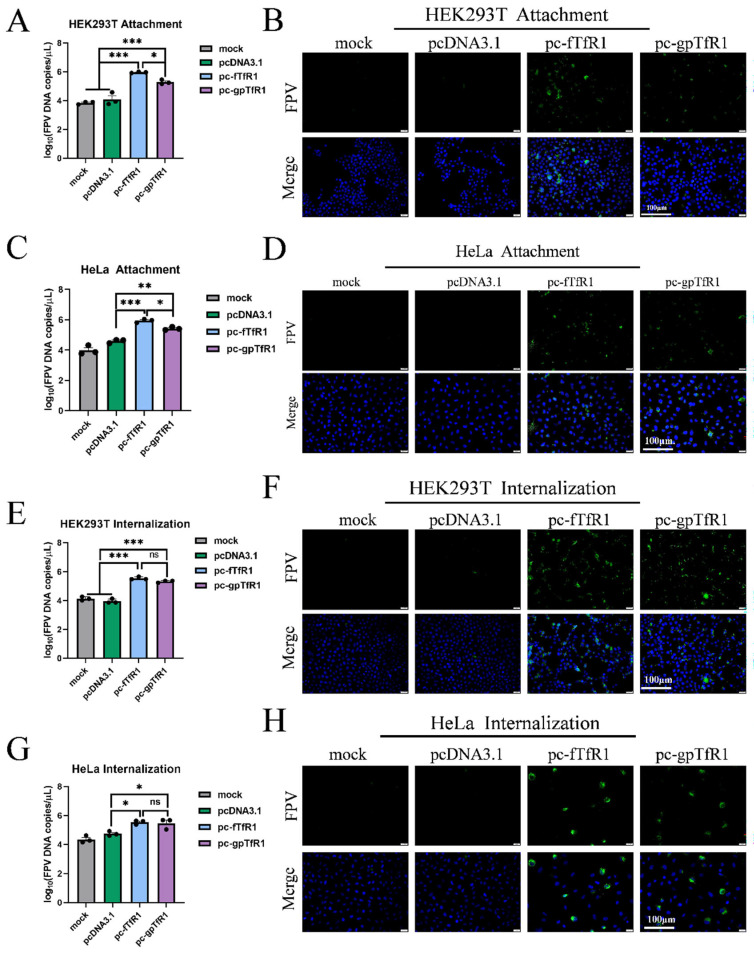
Effect of gpTfR1 expression on the internalization stage of pFPV-sc adsorption. (**A**,**C**,**E**,**G**) FPV copy numbers detected using qPCR. (**B**,**D**,**F**,**H**) The number of infected cells detected by indirect immunofluorescence assay. (Blue: nucleus; Green: FPV; ns: not significant; * *p* < 0.05, ** *p* < 0.01, *** *p* < 0.001).

After confirming the influence of gpTfR1 on viral replication, the effect of gpTfR1 on the entry of pFPV-sc into cells was further studied. By measuring viral copy numbers and performing indirect immunofluorescence experiments, we determined whether gpTfR1 expression affects specific stages of pFPV-sc binding or endocytosis. The results showed that viral copy numbers in both fTfR1 and gpTfR1 groups increased significantly during the virus adsorption stage. Concurrently, compared with the fTfR1 group, the viral copy number in the gpTfR1 group was slightly lower (Figure 4A,C). In the meantime, obvious fluorescence signals were observed in the fTfR1 and gpTfR1 groups via indirect fluorescence immunoassay, whereas no fluorescence signal was observed in the control groups (Figure 4B,D). At the viral internalization stage, the viral copy numbers in the fTfR1 and gpTfR1 groups increased significantly (Figure 4E,G), while no significant difference was observed between the fTfR1 and gpTfR1 groups. Contemporarily, through the detection of fluorescence signals via an indirect fluorescence immunoassay, obvious fluorescence signals were observed in the fTfR1 and gpTfR1 groups, whereas minimal or no fluorescence signal was observed in the control groups (Figure 4F,H). These results indicate that the expression of fTfR1 and gpTfR1 enhances virus attachment and internalization, and that gpTfR1 has a lower effect on increasing virus copy numbers at the virus adsorption stage than fTfR1.

### 3.5. pFPV-sc Infection in Unsusceptible Cells Requires gpTfR1 Expression

**Figure 5:**
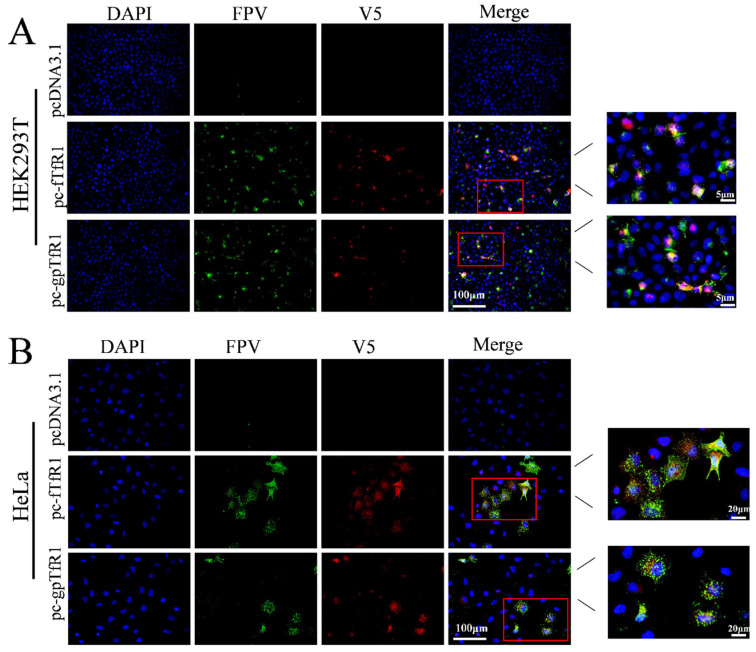
Immunofluorescence colocalization of gpTfR1 with pFPV-sc. (**A**) Colocalization of gpTfR1 and FPV in HEK293 cells. (**B**) Colocalization of gpTfR1 and FPV in HeLa cells. (Blue: nucleus; Green: FPV; Red: V5 tag).

To further validate that pFPV-sc infection and gpTfR1 expression occur in the same cell post-transfection, the subcellular localization of gpTfR1 and pFPV-sc was analyzed during the early stages of pFPV-sc infection. The results showed that virus infection (green) primarily colocalized with recombinant gpTfR1 (red), indicating that an early virus infection stage correlates with gpTfR1 expression (Figure 5). A similar pattern was also found with fTfR1 expression. Taken together, our observation demonstrated that the gpTfR1 association plays an important role in the process of pFPV-sc infection, which could serve as an initial and necessary step for FPV cross-species transmission to the giant panda population.

## 4. Discussion

Most virus infections begin by binding to specific cell receptors and the uptake of the virus into cells through receptor-mediated endocytosis [16]. This process is controlled by multiple factors, including the characteristics of virus-binding receptors, signal transduction, and endocytic properties, as well as the affinity of the virus for the receptor and the structural characteristics of interactions under certain conditions. Parvoviruses bind TfR1 to enter cells through clathrin-mediated endocytosis, which is then transported via the endosomal pathway [17,18]. Studies have shown that certain viruses can bind to TfR1 with different binding levels and affinities within different hosts [19,20]. For example, although the expression levels of TfR1 in feline and canine cells are similar, the binding and endocytosis levels of feline cells to canine parvovirus (CPV)-2 and CPV-2b capsids are 3.5 times and 5 times higher than those of canine cells, respectively, which might be due to the relatively lower binding affinity of CPV capsids for canine cells [17]. At 37 °C, there is a difference in the binding levels of feline and canine TfR1 to CPV-2 capsids, and cells expressing fTfR1 show a higher level of binding and uptake. In the present study, we showed that expression of fTfR1 and gpTfR1 had a significant promoting effect on viral infection at both the virus adsorption and internalization stages. In addition, at the virus adsorption stage, compared to fTfR1, gpTfR1 exhibited a lower infection level, but the internalization levels were similar. The difference in replication levels between fTfR1 and gpTfR1 is likely due to the higher affinity of the virus against fTfR1, where the probable differences in the apical domains between fTfR1 and gpTfR1 may play an essential role, as with the host specificities of CPV and FPV [19,20]. Concurrently, the colocalization of gpTfR1 and pFPV-sc infection was analyzed by immunofluorescence colocalization experiments in the early stage of infection, and it was found that there was an aggregation of pFPV-sc at the location of gpTfR1. In conclusion, gpTfR1 may first promote infection by virus association, followed by promoting viral replication at the initial stage of viral infection.

Parvoviruses display restricted host specificity. For instance, studies have shown that FPV cannot bind to canine TfR1, which is only 12% different from fTfR1, indicating strong host specificity [12]. The amino acid homology between fTfR1 and gpTfR1 is approximately 87.66% [8]. FPV should also have a host barrier against gpTfR1. However, in natural ecosystems, the phenomenon of FPV cross-host infection of giant pandas has been observed. Therefore, using fTfR1 as a positive control, we explored the receptor for FPV infection in giant pandas. The results of this study showed that gpTfR1 expression in non-susceptible cells has a function similar to that of fTfR1 and can significantly promote the replication of pFPV-sc. This effect may have occurred at an early stage of viral infection, indicating that gpTfR1 may play an important role in the life cycle of FPV, possibly by promoting viral entry into cells. Although fTfR1 may be similar to gpTfR1 in some respects, there are still differences between them, and further research is needed to verify the exact role of gpTfR1 in FPV infection. FPV, which initially only infected felines, has evolved to cause a CPV pandemic in canines as a host-range variant through several key amino acid changes in the VP2 capsid protein [19]. Currently, they are no longer limited to felines and canines but can infect a wider range of animal groups, including Ursidae and primates. This trend has prompted consideration regarding the continuous evolution of FPV and raised questions about its potential to infect humans through mutual adaptation to the host TfR1. The present study found that pFPV-sc caused sporadic infections in human HEK293T and HeLa cells, suggesting a potential risk of cross-species transmission of FPV. Future research is required to further explore the evolutionary path of FPV and its interaction with the host to comprehensively assess its potential impact on human health and implement corresponding prevention and control measures.

In conclusion, gpTfR1 was used in the present study as a hypothetical surface receptor for virus–host binding, and the mechanism of FPV cross-species infection in giant pandas was investigated. These results showed that gpTfR1 is the cellular receptor for FPV infection in giant pandas and can promote FPV replication. This discovery is expected to provide a theoretical basis for the future development of antiviral intervention strategies and drugs that can prevent FPV infection from giant pandas.

## Figures and Tables

**Table 1:** Amplification primers and target fragments.

Primer	Sequence (5′-3′)	PCR Products/(bp)
F81-fTfR1-F	aaaaaGGATCCATGATGGATCAAGCCAGAT	2310
F81-fTfR1-R	aaaaaCTCGAGAAACTCATTGTCAATATCCCA	
TfR1-mRNA-F	TGGCTGTATTCTGCTCGTGG	189
TfR1-mRNA-R	GCCCCAAAAGATATGTCGG	
F81-RPL17-F	CTCTGGTCATTGAGCACATCC	123
F81-RPL17-R	TCAATGTGGCAGGGAGAGC	
293-βactin-F	CAAAAGGCGGGGTCGCAAT	108
293-βactin-R	CGACGATGGAAGGAAACACG	
Hela-PPIA-F	GAGGAAAACCGTGTACTATTAGC	86
Hela-PPIA-R	GGGACCTTGTCTGCAAAC	
VP2-qPCR-F	ACGGGTACTTTCAATAATCAGAC	179
VP2-qPCR-R	AATATCATCTAAAGCCATGTTTC	

Note: Primers F81-fTfR1-F and F81-fTfR1-R were used to construct the expression vector. Primers TfR1-mRNA-F, TfR1-mRNA-R, F81-RPL17-F, F81-RPL17-R, 293-βactin-F, 293-βactin-R, Hela-PPIA-F, and Hela-PPIA-R were used to detect the mRNA transcription level of TfR1. VP2-qPCR-F and VP2-qPCR-R were used to detect FPV copy number.

## Data Availability

All data shown in this manuscript is assessable upon reasonable request.
